# The Scholar in Professional Life1An address to the graduating classes at the 58th annual commencement of the University of Buffalo May 3, 1904.

**Published:** 1904-06

**Authors:** Frank Hyatt Smith

**Affiliations:** Buffalo, N. Y.


### BUFFALO MEDICAL JOURNAL.

VOL. XLIII.—LIX. JUNE, 1904. No. 11

### ORIGINAL COMMUNICATIONS.

### The Scholar in Professional Life.[1]

By FRANK HYATT SMITH, M. A., Buffalo, N. Y.

*Members of the Graduating Classes:* I deeply appreciate the honor conferred upon me by those who have invited me to address you on this interesting occasion. To this hour you have long looked forward and for it you have steadily prepared; and now in the presence of your honored instructors and of this great assembly of friends and observers, you, as graduates of the University of Buffalo, face with eagerness and enthusiasm the activities of the world in which you are soon to play an important part. He must be dull, indeed, whose mind in such an hour is not thrilled with subtle influences and mysterious emotions. The busy age, the manifold needs, the preservation of society, the transmission of character, the alleviation of pain, the permanence of truth,—these themes are both inviting and rewarding. It is, however, with a peculiar sense of obligation, that I have chosen as the topic of my address, The Scholar in Professional Life.

It is the glory of Francis Bacon, the founder of empirical philosophy, that, in his catalogue of sciences, he gave the highest honor and place to those that have to do with the physical well-being of man. You will leave to others the subtilties of learning, the speculations of dogmatists, the measurements of distant worlds; it will be your great province to lengthen and maintain life, to dispel suffering, to extinguish disease, to overcome contagion, to war with pestilence, to compound remedies, to devise sanitation, to improve hospitals, to relieve the injured, to amend tenements, to make constant researches into the origin of pain,

1. An address to the graduating classes at the 58th annual commencement of the University of Buffalo May 3, 1904.

the structure of the body, and the cure of human ills. I heartily congratulate you on the opportunities before you. There was a time when medicine and surgery and pharmacy and dentistry were fettered and under opprobrium. The greatest discoverers to whom you owe your proficiency, men of whom the world was not worthy, have been satirised and ostracised and persecuted; they endured the bigotry and ignorance and tyranny of those who knowing nothing fancied they knew all.

> "How few think justly of the thinking few,
> How many never think who think they do."

But the world moves and your scientific inquiries will be free as the air and wide as the sea. Each new day startles us by its possibilities; the chemist is everywhere sought; minerals hitherto unknown and vegetables before despised, are yielding medicines for men's sickness, tonics for their weakness, balms for their pains. The state itself is assisting to eliminate, if possible, the ravages of cancer and consumption, and lately listened with respect to the appeal of one of your noted instructors for the maintenance of a fund for this sublime end. The paradox of yesterday is the commonplace of today. We have new alchemists who transmute radium into helium, extract from the clouds vitalising forces, and in the solitude of the laboratory delve into the mysterious cellular relations of life. Dentistry has made a marvelous advance in the last twenty-five years and American practitioners lead the world. It was an American dentist who assisted the first lady of France in her dire misfortune. The care and preservation of the teeth is now of universal importance, and the army must have its dental corps. To explain the origin of a tooth formation, to devise a dental improvement, to arrest decay,—these are quite as noble as to tunnel a mountain or bridge a stream. To Victoria's physician we owe her matchless services; to King Edward's surgeon the British nation owes its present ruler. And in the hour of our own calamity, when a great leader was stricken by the assassin, it is the honor of this University that to the members of its Faculty the distinguished sufferer was at once entrusted for surgical aid. "Peace hath her victories no less renowned than war."

Much is said in our time of the strenuous life. Patriotism is too frequently estimated by sword and shell. The greatest forces are silent. The flowers offer no advertisement and the sun beats no drum. The world contains no higher patriot than the obscure and faithful physician, removed from the struggle of commerce, disdaining rest and sleep, the willing servant of the unfortunate and depressed, hiding his own sorrows, bearing others' burdens, and bringing health and good cheer to hundreds who forget what is his due. His close ally is the accurate pharmacist, with a pitying eye for the delusions of men, busy at the prescription counter when society is wasting itself in dissipation, and exact in his selections and weights when a single error would cost a life. The teacher fills the brain with ideas, but the dentist fills the mouth and makes the orator and singer possible. Nor dare I omit a tribute to the trained nurse, whose sagacity, serenity, courage and devotion have made her a necessity in the sickroom and in the hospital, on the field and in the camp. Policy asks is it popular, avarice asks will it pay, society asks is it fashionable, duty asks am I needed. A few rain drops frighten a congregation; a pestilence attracts a doctor. People will weep over a melodrama and then run away from a train wreck; the intrepid surgeon and the undaunted nurse, with instruments and bandages hurry to the scene of horror, and there remain until the last sufferer is relieved. We have too many churches and too few hospitals.

The Good Samaritan, who saw the stranger on that Jericho road, ran to him, poured in oil and wine and bound his wounds, placed him on his own beast, and carried him to an inn,—he is your model for all time. No man asks his creed or his politics or the cut of his coat. He did, while others only gazed. He has neither name nor title in the record. But he was the first emergency hospital and will be revered when Wall street is forgotten. Your watchword is duty, the noblest word in any language, and fast becoming lost from the average lexicon. I urge you, by example and precept, by the traditions of your great profession, by the memories of your instructors, to love your work for humanity's sake, and, like the Great Physician, go about doing good. Your reward must come from within.

There can be no adequate compensation for a life saved. Milton received less than a hundred dollars for the great epic, but it seems to last. Pure disinterested labor is now rare. I wish that you might find an antitoxin for the fever for gold that possesses the nation. Do all you can to recall the old Spartan simplicity of life and manners. Send your patients out into the woods, where trusts and skyscrapers and trolleys are unknown. In the zeal for culture we have forgotten our courtesy, we can name the latest novels but not the ten commandments, men and women go from fad to fad in feverish haste, and suddenly drop at an age when their parents were ripe.

Each century has its distinguishing characteristic. We live in a period when every theory finds its advocate and the wildest delusions prevail. Let a saffron cloaked apostle of some East

Indian cult from beyond Madras, with flowing locks and sandalled feet, suddenly appear in the American Athens, announcing that he would give away a vial of the river of paradise, and confer immortality on all who would pay his extortionate rates, the streets would be thronged with eager disciples and female clubs would be emptied to crowd his lectures. Esau sold his birthright and the soberest men and women are willing to part with their endowment of common sense for the merest bauble. Only horses and dogs refuse patent nostrums. I mention this singular tendency of our times because you will have to meet it in your work. It is the outcome of an age of inquiry. There are hundreds who deny the existence of matter and disease. With such all argument is futile.

To those who refuse all curative agencies and disdain the healing properties of the drugs with which the Creator has filled the earth, it should be sufficient to quote Friar Laurence in the play of Romeo and Juliet, as he gathers his medicines in the early morning:

> "I must upfill this osier cage of ours,
> With baleful weeds and many juiced flowers:
> Many for many virtues excellent,
> None but for some and yet all different:
> O, mickle is the powerful grace, that lies
> In herbs, plants, stones, and their true qualities:
> Within the infant rind of this small flower
> Poison hath residence and medicine power."

We might, indeed, paraphrase Shakespeare again and ask with genial Falstaff: "Can mind set a leg? No. Or an arm? No. Or take away the grief of a wound? No. Mind hath no skill in surgery then? No." The record of the creation gives us the first instance of the use of anesthetics, for before the Creator made woman from the rib of man, He put him in a deep sleep. When King Hezekiah was sick unto death they laid figs on him and he recovered; they did not appeal to mortal mind. The minister sees men at their best, the lawyer sees them at their worst, and the doctor sees them as they are.

You will need and I trust acquire a broad and catholic charity for people, who are largely as they are made. One failure of yours will be remembered more than ten successes. You will be summoned by those who do not need your services, and be attacked for telling such the truth. Often you will be dismissed for trivial reasons by persons whose tastes change with the wind. In all such crises bear yourselves with dignity and charity. Good wine needs no bush. If men like an impostor, let another have that unenviable name. Boy healers and hypnotic manipulators and mental transformers and immortality venders and absent-thought-current originators, like Judas, find their own place. There is nothing new under the sun, and all these were anticipated centuries ago. Dickens portrays such in the exquisite satire of Bob Sawyer and his partner. Could that unrivàlled master of fiction rise from his grave and devote one work to the elimination of all the quacks in the medical world, they would melt into air, thin air. I sometimes think that like mosquitoes they might be dispelled by coal oil, liberally used.

In all your development be modest. It was remarked of the great oculist, Cornelius R. Agnew, that he was so unassuming that he was often taken for his assistant. Simplicity is greatness. Humility is the portal of achievement. Bacon tells us that the kingdom of science, like the kingdom of heaven, must be entered in the spirit of a little child. The pompous politician and clergyman and lawyer and physician are alike repelling. "Knowledge is proud that he has learned so much: wisdom is humble that he knows no more." Five smooth stones from the brook slew the giant. Cultivate industry, integrity, economy, courtesy and skill, and you will surely win. Your skill will increase with years.

In the Norse legend, Thor the strong man went to the palace of the gods in the twilight land. There he boasted of his powers. The king, to test him, brought out a dwarf to wrestle with the stranger, and the dwarf threw him at the first round. Then he asked him to run a race with an old woman, and she reached the goal before he had gone a yard. Then he gave him a drinking horn to drain, but as fast as he drank its contents, it filled again. And at last he tried to eat a trough of meat but the trough was never empty. To the disconsolate Thor the king cried, "O! Thor, when you wrestled with the dwarf you wrestled with fate; when you raced with the old woman you raced with thought; when you sought to drain the horn you sought to drain the sea; and when you attempted to eat the meat you were trying to overcome the grave which is always filling." This should teach us the value of moderation. "In medio tutissimus ibis." Cultivate a judicious silence. You will be the custodians of the most sacred family relations. Never betray them. A gossiping doctor is an abomination. The Divine Physician was silent when praised, silent when blamed, silent when He wrought His cures, silent when all men talked. Silence conserves energy.

This is an age of specialists. The average in every department of human endeavor is rising so fast that more and more men are devoting all their time and talents to one work. I am not certain that this is an auspicious sign. The eye of the specialist is like the lens of the microscope, fine in its distinctions but narrow in its range. Emerson tells us that a man is the prisoner of his power, and that the farmer becomes a spade and the banker a coin and the clerk a ledger. You see the danger. It can only be corrected by wide reading and an active interest in the general life of men. If you give your attention to but one department of your science, do not let it master you. Everett said that Washington was a sphere, all points of which were equally distant from the center. Thorough cultivation now is very rare and the world is full of angular and fractional men. Edmund Burke said that he could learn something new from every man. Try to know something of everything and everything of something. Break up the monotony of your professional life by little excursions, side avocations; interest yourselves in the arts, give your spare time to the best reading.

To a physician, Dr. John Brown, of Edinburgh, we owe some of the finest stories in the world. To that great doctor and writer of the 17th century, Sir Thomas Browne, the world is indebted for that masterpiece of quaint philosophy and subtle thought, "The Religio Medici," which every doctor should own and read. It is a treasure house of information and delight. Keep up your acquaintance with the classics for they are the avenues to culture. Perhaps there may be a Weir Mitchell among you who shall produce a great novel in the years to come. Try to make utility rather than personal gain the end of your labors. Wherever one looks the aim of human endeavor seems to be the dollar. In Milton's great epic we read that Mammon was "the least erected spirit among those that fell from heaven," and I urge you by your example to show the world that pure disinterested philanthropy still exists. It is the glory of the medical profession that with rare exceptions its members have wrought for general good and not for individual gain.

In these sordid days it is to the physician if to any one that men look for sacrifice and duty at any cost. Yet the laborer is worthy of his hire and the state should make the physician secure of his compensation. Chaucer in describing the doctor of his day gives us the subtle touch:

> "For gold in phisik is a cordial,
> Therfore he lovede gold in special."

Ninety-five per cent. of the people judge you for what you appear and five per cent. judge you for what you really are. Therefore cultivate neatness in dress at all times. Careless manners and untidiness are bars to recognition. Be content to wait patiently for success. I knew of an American dentist who began his practice in a German city. He secured an office on a prominent street and waited. Day after day he went down and no caller came. Months passed and he was in despair when one morning to his surprise a member of a noted family rang his bell and entered. Without a tremble he took his blank book, scanned its vacant pages and said: "Madame, I shall have to put your engagement ten days ahead; can you come at that time"? That man was a hero indeed. The tide turned and now he is rich. A. T. Stewart, the great merchant of New York City, in his early days had a very small business. He induced a wealthy customer to help him by allowing her carriage to stand at his door whenever she was in his vicinity. A word to the wise is sufficient.

I am not sure that your profession is without the jealousies that generally prevail wherever competition is strong. You cannot afford to speak ill of any man. When Edmund Burke was attacked in Parliament he replied, "no gentleman will insult me; no other can." All through life expect unjust and critical comment from those who differ from you in training and belief. Remember the race horse; keep trotting and the mud will dry and drop off. We waste much energy slapping mosquitoes. You are to battle with disease and not with petty slanders. Hold your minds open to all discovery even if you are compelled to recast your early views. Lord Kelvin says that it does not now become a man of science to doubt the possibility of anything.

"Be not the first to cast the old aside:
Nor yet the last by whom the new is tried."

The world is divided into two great classes: those who receive only what is new and those who reject what is old. Between these are the safer men who prove all things and hold fast to that which is good. Unfortunately, we are all bound by the chain of prejudice and our emancipation is slow.

That great philosopher Sam Weller said: "He is a man of science which is the mother of invention and knows no law." Men of science are not always models of toleration and liberality. The mathematician said that the Paradise Lost did not interest him for he could not multiply it; and the rhetorician replied that he despised algebra for it would not rhyme. Paley assures us that nothing has so retarded the progress of the human race as this principle,—contempt prior to examination. The fly on Saint Peter's dome sees but the smallest segment of the arc. "Behold we know not anything." Newton and Kepler were abashed in contemplation of the realms of knowledge they had not explored; it would not be difficult for us to find men who eagerly report each discovery that they make, or think they make,—to the press.

I do not think I transcend the limits of this address if I ask you to cultivate a reverence for the human body. Familiarity too often breeds contempt. The chemist sees only perfume in a flower, the lumberman notices only the girth of the tree, the electrician cares only for Niagara as a water franchise, and the anatomist is tempted to regard the body as material for operation and experiment. "We touch the Creator when we touch the body that He has made," said Richter. Coarse and brutal references and jests are least becoming in those who, most of all men, know the capacities and possibilities of the wonderful temple that we inhabit. When John Hunter was taunted by a professor for his ignorance of Greek, he said to his student, "go tell that man that I can teach him on the dead body what he never learned in any language living or dead." Delicacy and tenderness may not always be possible for you, but when they are remember to make such prominent. Roughness is no essential to success anywhere. The age is so intense and nervous that your function will be that of the balance in a watch, to ensure steadiness and tone.

I congratulate the ladies who have mastered their branches and are to demonstrate their ability with men. When Wendell Phillips demanded the right of women to enter the professions, thirty years ago, he was ridiculed in Boston. But he was a prophet as well as a reformer, and today we see Portia at the bar, Helena in the sick room, and Ida in the college. As Dante estimated his successive steps in Paradise by seeing the face of Beatrice in ever lovelier beauty, so the world now measures its advancement, not by inventions nor by cities, but by the force and grace and power of woman, once man's toy and drudge. Dryden tells us that a laugh is a good thing at any rate, and a straw if it tickle a man becomes an instrument of happiness. Your profession, more than all others, will require cheerfulness. A smile is often an excellent prescription and geniality is tonic to the sick. We allow other men to croak with their pessimistic plaints, but we expect the doctor to be the cricket on every hearth. What a robust optimism, what infinite stores of humor, what a perpetual fount of happiness he must be compelled to carry or assume. All draw upon that bank, yet it never suspends. And in times of great scourge, when society flees, and business closes its doors, and the court and church and school are empty, you will steadily pursue your daily and nightly round, the hope of the stricken and the friend of the dying, defying weather and pestilence and contagion, and some of you may be martyrs unsung in verse and unhonored in prose. This gives to your work its crowning grandeur. He that saveth his life shall lose it. The aggregations of capital, the achievements of commerce, the profits of banking, the wiles of the statesman, the possessions of the miser, the contentions of nations—all terminate on self and fade as the grass: while a single such hero as William MacLure, battling with disease and death among the simple farmers of the Glen, to whom the sight of his white mare wakened courage and hope, and at last borne to his grave through drifts of snow by stalwart men bowed in tears,—writes his memory in the human heart and is mourned by hundreds who say: "I was sick and he visited me."

To relieve human misery,—this is the aim of the scholar in medical life. Behind the foreground of your professional skill there must be the background of your personal character. Shadow is proportional to height. The foundation of all art is moral. Space has three dimensions; life has four,—length of days, depth of conviction, height of aspiration, and breadth of sympathy. When these are symmetrical you have a man. Let not your left hand know what your right hand does. Proficiency needs no advertisement. Let it be said that you are true men and women of purity, sincerity and elevation. Then your influence will be as pervasive as the air and as healthful as the light. You go forth from a university rich in its past and auspicious in its future, including many names justly known and widely honored. It looks to you to perpetuate its fame. Be loyal to your profession, to your instructors, and to yourselves. Then, whether your career be long or short, prominent or secluded, you will have the consciousness of a life well spent which is its own sufficient reward.

20 Huntington Avenue.

